# Challenges in Cerebral Venous Thrombosis Management—Case Reports and Short Literature Review

**DOI:** 10.3390/life13020334

**Published:** 2023-01-26

**Authors:** Florentina Cristina Pleșa, Alina Jijie, Gabriela Simona Toma, Aurelian Emilian Ranetti, Aida Mihaela Manole, Ruxandra Rotaru, Ionuț Caloianu, Daniela Anghel, Octaviana Adriana Dulămea

**Affiliations:** 1 Department of Neurology, “Dr. Carol Davila” Central Military Emergency University Hospital, 134 Calea Plevnei, 010242 Bucharest, Romania; 2 Department of Preclinical Disciplines, “Titu Maiorescu” University, 031593 Bucharest, Romania; 3 Department of Radiology, “Dr. Carol Davila” Central Military Emergency University Hospital, 010242 Bucharest, Romania; 4 Department of Endocrinology, “Dr. Carol Davila” Central Military Emergency University Hospital, 010242 Bucharest, Romania; 5 Department of Medico-Surgical and Prophylactic Disciplines, Faculty of Medicine, “Titu Maiorescu” University, 031593 Bucharest, Romania; 6 Department of Internal Medicine, Central Military Emergency University Hospital, 010242 Bucharest, Romania; 7 Neurology Department, Fundeni Clinical Institute, 022328 Bucharest, Romania; 8 Neurology Department, Carol Davila University of Medicine and Pharmacy, 050474 Bucharest, Romania

**Keywords:** cerebral venous thrombosis, pregnancy, seizure, postpartum, hemorrhage, depression

## Abstract

Cerebral venous thrombosis (CVT) is a rare type of stroke, with a complex clinical presentation that can make it a diagnostic challenge for the swift initiation of anticoagulation. When a hemorrhagic transformation is added, therapeutic management becomes even more complex. We describe a series of four cases, aged between 23 and 37 years old, with cerebral venous thrombosis. They were admitted to our clinic between 2014 and 2022. All cases presented significant challenges in either diagnostic, therapeutic or etiologic evaluation, at different stages of the disease. Late complications such as epilepsy or depression and other behavioral disorders represent long-term sequelae for the patient. Therefore, through its late complications, CVT is not only an acute disease but a chronic disorder with long-term follow-up requirements. The first case of the series is of a postpartum woman with focal neurological deficit caused by CVT with hemorrhagic transformation that presented multiple thrombotic complications and severe depression. The second case is of a man with extensive cerebral thrombosis who developed bilateral papillary edema under therapeutic anticoagulation treatment. The third case is of a woman with bilateral cavernous sinus thrombosis who later developed depressive disorder and focal seizures. The fourth case is of a pregnant woman in the first trimester presenting with a steep decline in consciousness level secondary to deep cerebral vein thrombosis requiring intensive care and subsequently developing a memory disorder. For a long period of time, due to being underdiagnosed, few things were known about CVT. Nowadays, we have all the tools to diagnose, treat, and follow up cases of CVT.

## 1. Introduction

Cerebral vein and dural sinus thrombosis (CVT) is one of the least frequent types of strokes, although it represents one of the most important causes of stroke in young adults, with an average age of 37 years old [1,2]. 

Epidemiologically, CVT has an annual incidence of 1.16 to 2.02 cases per 100,000 and is more frequent in females than males, with a ratio of 3:1 [2] due to the association with the use of oral contraceptives, pregnancy, and postpartum period. There are other prothrombotic factors involved such as thrombophilia, malignancy, and localized or systemic infections.

Thrombosis of the veins or venous sinuses, through the alteration of the blood–brain barrier and the reduction in CSF absorption, causes an increase in intracranial pressure (ICP). In 26% of patients, this can be the only manifestation of the onset of the disease, misguiding the diagnosis [2]. Sometimes the symptoms are complex and non-specific and further complicate the diagnosis which can be established using cerebral CT venography or MRI venography with contrast if MRI imaging is rapidly available [3].

The most common locations are at the level of the superficial venous sinuses and, frequently, several sinuses are involved. The treatment of acute CVT is primarily aimed at stopping the progression of the thrombus, restoring venous blood flow, and preventing thrombotic relapses.

With its myriad of clinical manifestations and challenging imaging diagnosis, CVT requires careful management, particularly in the treatment strategy. Overall, CVT has a favorable prognosis. However, it may sometimes be complicated by various, yet rare, coexistent pathologies such as focal deficits or altered state of consciousness and, in the long term, epileptic seizures or mental disorders [4].

In this article, we present four different cases that represented a diagnostic and therapeutic challenge in terms of symptoms, etiology, evolution, and complications.

## 2. Results

### 2.1. Case Presentation 1

A 23-year-old obese woman developed right-arm paresthesia followed by ipsilateral arm and leg weakness, as well as right-sided hemi-face paresthesia 4 days post-partum (cesarean delivery), complicated by pulmonary thromboembolism and deep vein thrombosis in the acute phase, long-term sequelae of moderate depression, and one severe episode of major depression, with an autolytic attempt 2 years later.

Regarding the pregnancy, she was a primigravida, with no miscarriages (spontaneous or medical) and no history of oral contraception use or smoking. The pregnancy had not been complicated, and the delivery by cesarean section was due to prolonged labor; subsequently, the mother and child took 4 days of antibiotics because of meconium contaminating the amniotic fluid. Her medical and family history were unremarkable.

Four days later, she was transferred to our clinic due to the development of neurological symptoms. The neurological exam showed a conscious and cooperative patient with no signs of meningeal irritation or right lateral homonymous hemianopsia and no other cranial nerve involvement, with right hemiparesis of 3/5 brachial on the Medical Research Council Scale (MRC) and 4/5 MRC crural, as well as hyperesthesia, brisk deep tendon reflexes, and a positive Babinski sign, all on the right side.

**Figure 1:**
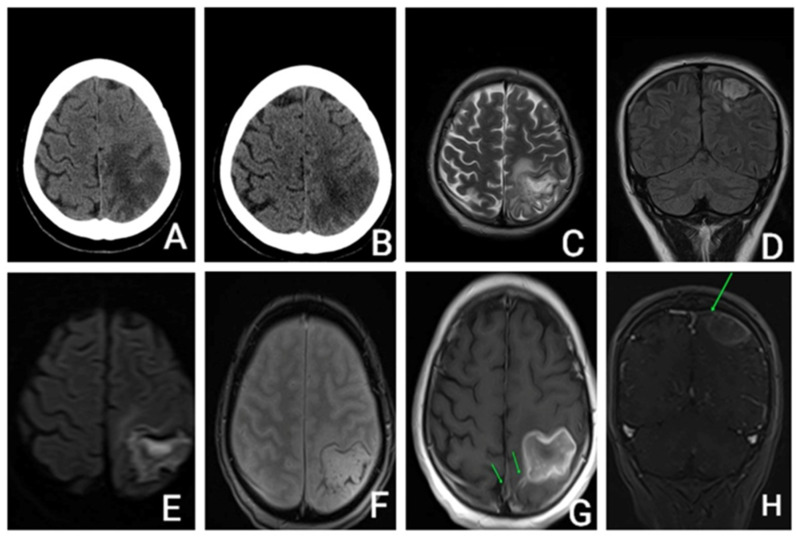
(**A**,**B**)—Unenhanced CT axial: (**A**)—acute parietal stroke with hemorrhagic transformation; (**B**)—9 days apart—Unenhanced head CT axial: subacute parietal stroke with hemorrhage resorption. (**C**–**H**): Brain MRI with and without contrast enhancement: (**C**)—T2 axial, (**D**)—FLAIR coronal, (**E**)—DWI axial, (**F**)—T2* hemo axial, (**G**)—T1 axial after i.v. contrast, (**H**)—T1 coronal after i.v. contrast. Ischemic area with hemorrhage transformation, hemosiderinic deposits mostly in periphery, central restricted diffusion and peripheral enhancement, filling defects of the sagittal venous sinus and of a cortical vein towards the affected left parietal area. CT—computer tomography; T1—weighed image; T2 weighted image; FLAIR—fluid attenuated inversion recovery; DWI—diffusion weighted imaging; T2* hemo—T2 weighted sequence.

She underwent an unenhanced cerebral CT that revealed a parietal hypodensity with hemorrhagic transformation. (Figure 1).

Routine bloodwork at admission revealed a hypochromic microcytic anemia (iron deficiency) and an inflammatory syndrome (associating fever); microbiological examination of vaginal secretions came back positive for *Klebsiella pneumoniae* and *Escherichia coli.*

Therefore, she was started on antibiotics and supportive treatment. Throughout the course of the investigations, the patient was clinically stable. 

The imaging aspect and the clinical picture of the patient made a positive diagnosis difficult. Due to her age, we had to look for the more common etiologies of stroke in a young patient. She underwent transthoracic and transesophageal echocardiography, in search of a patent foramen ovale or endocarditis, taking into account the recent history of infection. Both suspected diagnoses were disproven. We also looked for a collagen vascular disease (such as systemic lupus erythematous, antiphospholipid syndrome, ANCA-associated vasculitis, and large-vessel vasculitis), syphilis, and atrial fibrillation (24-h Holter monitor), but none of them came back positive. No arterial dissection or stenosis/occlusion was found at the cervical Doppler ultrasound and a tumor pathology or arteriovenous malformation was discussed; all these etiologies were later disproved. 

The head CT scan, performed 9 days after admission, showed that the hemorrhage was in a resorptive stage. The diagnosis of CVT was then considered and contrast-enhanced cerebral MRI confirmed sagittal venous sinus thrombosis. 

In the meantime, the patient started complaining of left ilioinguinal and lower leg pain with minimal left-leg swelling. She underwent lower-limb Doppler ultrasonography which revealed left ilio–femoral–popliteal thrombosis. She was then immediately transferred to the cardiovascular intensive care unit and was started on continuous i.v. heparin and close neurologic monitoring was initiated. Considering that she also had a progressive thrombocytosis, from 321 k/μL to 619 k/μL (with the lab reference values 140–440 k/μL) and iron deficiency, the risk–benefit balance was in favor of anticoagulation. 

**Figure 2:**
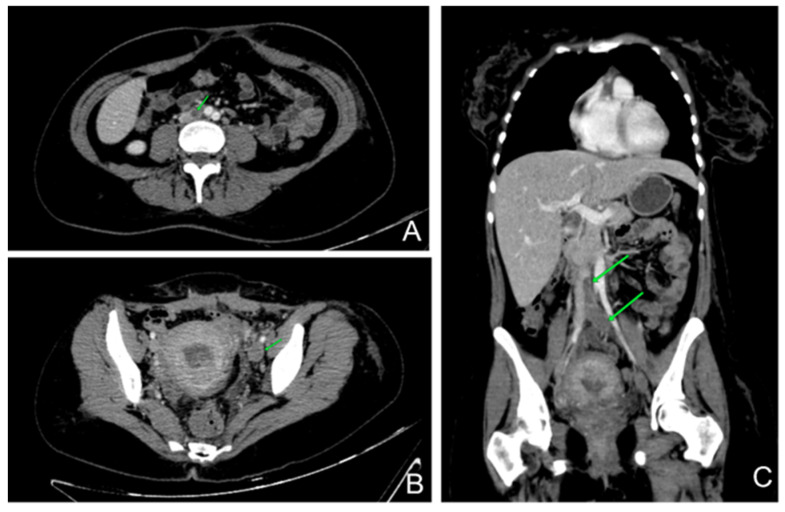
Contrast-enhanced thorax abdominal and pelvis CT: (**A**)—axial abdominal CT shows thrombus in inferior vena cava; (**B**)—axial pelvis CT reveals the extension of the thrombus in the left external iliac vein; (**C**)—coronal CT encompasses the length of the thrombus.

After six days under heparin treatment with therapeutic dose, she started to complain of interscapular pain and dyspnea with slight oxygen desaturation (SpO_2_ 94%). She immediately underwent a chest–abdomen–pelvis contrast-enhanced CT that revealed moderate pulmonary embolism and a left inferior vena cava thrombosis (Figure 2). Having a high risk of cerebral bleeding and extensive deep vein thrombosis, the placement of a filter on the inferior vena cava was considered.

She was hospitalized for 40 days and discharged with a positive clinical and paraclinical outcome, with an improvement in motor deficits to 4/5 MRC right hemiparesis, and long-term acenocoumarol anticoagulation with target INR between 2 and 3.

The thrombophilia test, partially performed one year after the acute event, revealed positive results: antithrombin III deficit, low activated protein C resistance, and hyperhomocysteinemia, thus making her a candidate for chronic anticoagulation.

**Figure 3:**
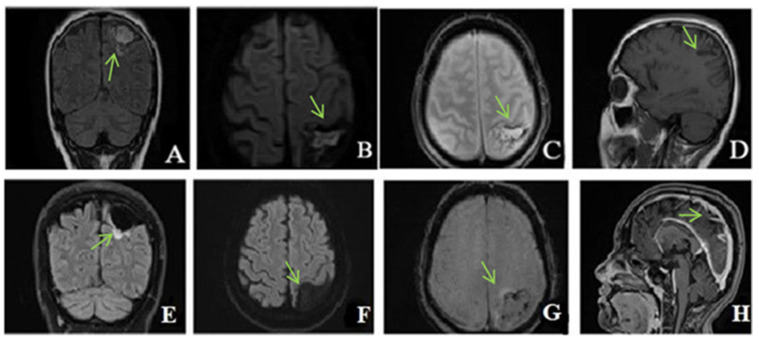
(**A**–**D**): Unenhanced brain MRI (at 3 months): (**A**)—Fluid attenuated inversion recovery (FLAIR) coronal, (**B**)—Diffusion-weighted imaging (DWI) axial, (**C**)—T2* hemo axial, (**D**)—T1 sagittal: Chronic ischemic left parietal lesion with hemorrhagic transformation partially resolved. E-H: Unenhanced and enhanced brain MRI (at six years): (**E**)—FLAIR coronal, (**F**)—DWI axial, (**G**)—T2* hemo axial, (**H**)—T1 sagittal after i.v. contrast administration: Chronic ischemic left parietal lesion with slightly peripheral gliosis, without restricted diffusion and minimal hemosiderin peripheral deposits, without filling defect of the veins and venous sinuses.

Close monitoring was performed after discharge, clinically, biologically, and radiologically. Three months after the CVT, she had completely recovered all her motor functions with the use of neurorehabilitation therapy. However, she developed symptoms of depression, for which she started receiving antidepressant medication. The brain MRI (Figure 3) showed the lesion as being chronic with the hemorrhage partially resolved at three months, with an aspect of organizing hematoma.

Three years later, she underwent another brain MRI (Figure 3) that showed a chronic ischemic left parietal lesion with partial peripheral gliosis and peripheral hemosiderin deposits. 

However, her depression advanced to moderate, having had one severe episode. Following this event, she was closely managed psychiatrically with antidepressant treatment and psychotherapy. The depression was kept under control, with no further major or moderate episodes. 

A follow-up brain MRI at six years showed the chronic lesion with no filling defect of cerebral veins and sinuses. 

We also performed complete thrombophilia tests, including Factor V G1691A (Leiden), Factor V H1299R (R2), MTHFR C677T, MTHFR A1298C, Factor XIII V34L, PAI-1 4G/4G, Factor II G20210A and Endothelial Protein C Receptor (EPCR) A1/A2 coupled with serology for: Ac anti-cardiolipin IgG and IgM, Ac anti-Beta2 glycoprotein 1, Ac-anti phospholipid IgG and IgM, lupus anticoagulant, homocysteine, and protein C and S activity. The results came back positive for Factor V Leiden heterozygous and PAI-1 homozygous, having thrombophilia with a medium-to-high risk, with the subsequent need for chronic anticoagulation. She is still under acenocumarol therapy at the indication of the hematologist. She is no longer in need of antidepressants, while still attending her weekly psychotherapy. 

### 2.2. Case Presentation 2

A 37-year-old overweight male patient with no past medical history presented with severe diffuse headache that started several days prior. The headache was refractory to usual analgesic medicine and got progressively worse. His wife recalled an episode of disorientation and slurred speech disturbance that lasted for a short period of time and stopped spontaneously. Upon arrival, vital signs were normal. The objective neurological examination was unremarkable. 

In what concerns the laboratory blood testing, inflammatory markers had increased values. The ECG showed bradycardia at the beginning, with normalization afterward.

**Figure 4:**
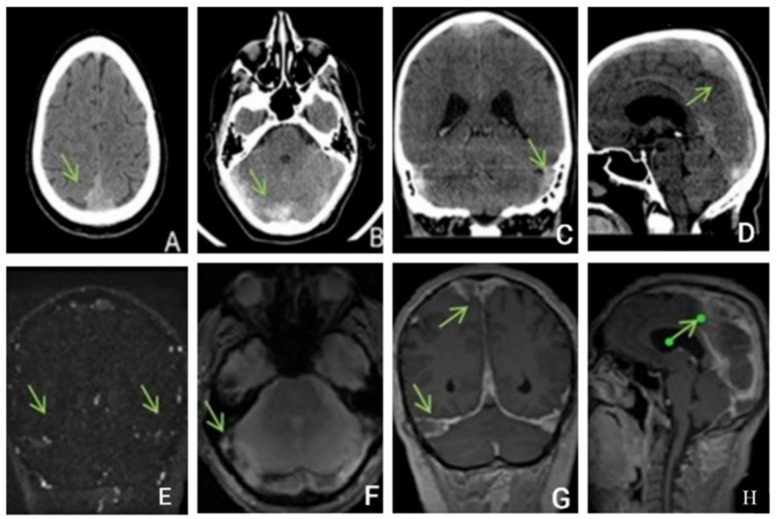
Unenhanced CT: (**A**,**B**)—axial, (**C**)—coronal, (**D**)—sagittal exhibits hyperdensity of the venous sinuses (sagittal superior and inferior, right sinus, transverse and sigmoid sinuses). (**E**–**H**) MRI, (**E**)—TOF (time of fligt angiography) venous coronal; (**F**)—T2*hemo axial, (**G**)—T1 coronal after i.v. contrast, (**H**)—T1 sagittal after i.v. contrast, expressed extensive thrombosis of all veins.

Native head CT performed at admission (Figure 4) showed increased dimensions of sinuses, and hyperdense content in the superior longitudinal sagittal sinus, the right sinus, and, contiguously, the inferior longitudinal sinus as well as in the right transverse sinus and partially the right sigmoid sinus. Additionally, a hyperdense appearance of the cerebellar tentorium raised the suspicion of a subarachnoid hemorrhage. The hypoattenuating brain tissue is suggestive of diffuse cerebral edema.

Blood samples were collected and tested for thrombophilia: lupus anticoagulant, antithrombin III levels, factor V Leiden levels and protein C and S levels did not have significant values. Afterwards, the anticoagulant treatment with unfractionated heparin in continuous perfusion was started. 

Further investigations highlighted increased homocysteine levels (17,1 μmol/l—with normal range < 10 μmol/L) and normal Vitamin B12 values (195 pg/mL, normal range: 187–883 pg/mL) for which the hematological consultation recommended anticoagulant treatment in combination with vitB12 and folic acid. 

Two days after admission, the patient underwent a contrast-enhanced brain MRI that showed extensive venous thrombosis at the level of the sagittal, transverse, and right sigmoid sinus with an increase in vascular caliber, absence of flow, and post-contrast filling defect. The association of fine bilateral high parietal subarachnoid hemorrhagic suffusions was also present, without constituted superficial parenchymal lesions. Inflammatory MRI signal changes at the level of the mastoids, frontal sinuses, and the ethmoidal cellular system were noticed, in keeping with pansinusitis. 

The oral anticoagulant treatment was started with acenocoumarol, with a target INR range between 2 and 3. Clinically, the patient was stationary, without new complaints or neurological signs. Another brain MRI was performed after 9 days. Compared to the previous examination, this was showing a slightly improved appearance, with the corresponding thrombi at the level of the superior sagittal sinus being reduced.

With a favorable clinical evolution and stable desired INR, the patient was deemed medically fit for discharge after 20 days. Long-term acenocumarol treatment was prescribed and follow-up imaging and hematologic review at three months were booked.

Ten days after the discharge, he presented again to the emergency department with headache, dizziness, visual acuity disorders, and diplopia. Upon admission, an ophthalmological examination was performed, revealing bilateral papilledema. INR was within therapeutic limits (INR = 2.66). Given the symptomatology, a new angio-MRI was performed that showed improvement in thrombosis compared to the previous examination.

After three days of associated antiedematous treatment, the patient was discharged again with no further complaints. 

The fluctuating symptomatology under anticoagulant treatment, in the context of an extensive cerebral venous thrombosis, in a patient with hyperhomocysteinemia recommends the continuation of the anticoagulant with clinical and imaging re-evaluation at one year.

At the one-year follow-up, the tests for thrombophilia were normal except for the maintenance of homocysteine at elevated values. 

MRI reassessment was also performed which revealed T2 and FLAIR hypersignal, but no DWI signal of the right subcortical frontal structures—a spot of ischemic gliosis. Furthermore, parietal FLAIR and flow void signal of the transverse sinuses was present in keeping with chronic thrombosis.

### 2.3. Case Presentation 3

A 36-year-old, overweight, female patient with medically controlled hypertension as the sole known cardiovascular risk factor came to the emergency department for intense headaches at the level of the right hemicranium, predominantly fronto-orbital, associated with dizziness. The symptoms were refractory to treatment and started three days before presentation. After the debut, the headache significantly progressed in severity, being also accompanied by dizziness, diplopia, mild photophobia, nausea, and inconstant paresthesia in the right limbs, along with a hypertensive spike. The neurological examination identified right external rectus muscle paralysis, mild palpebral edema, and redness at the level of the eyeballs, predominantly on the right side, alongside dysarthria. The patient complained of diplopia on frontal and bilateral horizontal gaze. Otherwise, the exam was unremarkable, without fever. The contrast CT Head performed was found to be normal.

The ophthalmological examination showed no signs of papilledema. On the same day, a lumbar puncture was performed, with unremarkable results.

**Figure 5:**
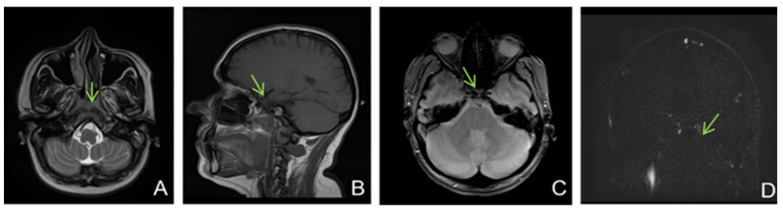
Non-enhanced MRI (at onset): ((**A**)—T2 axial, (**B**)—T1 sagittal, (**C**)—T2* axial, (**D**)—venous TOF coronal) shows unremarkable parenchyma and absence of flow in cavernous sinuses on TOF imagesFive months later, the patient came back to our clinic complaining of paroxysmal episodes of altered state of consciousness with language disorders such as verbal barrage followed by disorientation. An EEG was performed, showing a low-voltage background path, weakly modulated in spindles in the left derivations, reactive when opening the eyes, and rare isolated degraded peak-wave complexes, thus concluding that the patient was experiencing focal onset impaired awareness seizures; she was then started on antiepileptic treatment with oxcarbazepine 300 mg twice daily. She also was psychiatrically examined due to emotional lability confirming the diagnosis of depression and allowing antidepressant treatment to be started.

A brain MRI (Figure 5) showed flow asymmetry at the level of the cavernous sinuses, left more than right, coupled with fluid accumulation in the left mastoid cells, whilst cerebral parenchyma had morphology and signals within normal limits, thus establishing the diagnosis of bilateral (left > right) cavernous sinus thrombosis. The patient was started on continuous heparin infusion followed by treatment with acenocoumarol.

The six-month follow-up MRI identified persistent left maxillary sinusitis and cavernous sinus asymmetry but with an improved flow in the left one. Additionally, after 2 weeks of subcutaneous low-weight heparin, the panel for thrombophilia was requested and a protein C and S deficit was found, with the other tests (lupus anticoagulant, antithrombin III, factor V Leiden, IgM and IgG antiphospholipid antibodies, IgM and IgG anti-cardiolipin antibodies, IgM and IgG anti beta-2 glycoprotein I, p-ANCA, and c-ANCA) being within normal values. Thus, thrombophilia was diagnosed and the indication of lifelong anticoagulation established.

The patient continues to present a depressive disorder that improves under treatment and rare paroxysmal seizures with intermittent speech impairment. She was noted to be otherwise stable, with no further neurological sings.

### 2.4. Case Presentation 4

A 24-year-old woman in the seventh week of pregnancy was brought to the emergency department for a confusional syndrome onset in the morning of presentation.

As per her obstetrical history, she was a primigravida, with no miscarriages (spontaneous or medical) and no history of oral contraception use or smoking. Her medical history was unremarkable. Her sister had a full-term pregnancy, without complications and a spontaneous miscarriage in first trimester.

The patient presented an intense headache four days prior to the current admission, followed by dizziness and fatigue, for which she was investigated in an obstetrics–gynecology department and received symptomatic treatment.

She was admitted to our hospital with a heart rate of 98 bpm, in sinus rhythm, with a blood pressure of 180/100 mmHg, an oxygen saturation (SpO_2_) of 99%, and a temperature of 36.9 Celsius. 

The neurological exam showed a confused patient, with whom it was hard to cooperate. She was able to execute simple orders and had no signs of meningeal irritation, no cranial-nerve involvement, a bilateral Babinski sign, and was apparently without motor deficits.

Routine bloodwork at admission revealed slightly increased inflammation markers, without other abnormalities.

We decided to have an emergency MRI, but considering that the patient was pregnant, contrast was not used.

**Figure 6:**
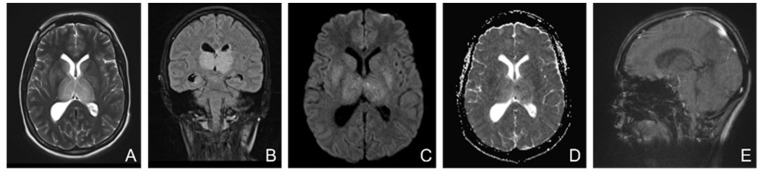
MRI without contrast enhancement ((**A**)—T2 axial, (**B**)—FLAIR coronal, (**C**)—DWI axial, (**D**)—ADC axial, (**E**)—venous TOF sagittal) shows hyperintensities of the thalamic nuclei on T2, FLAIR and DWI, with areas of restricted diffusion included and no flow in Galen’s vein, internal cerebral veins, straight sinus, and inferior sagittal sinus.

MRI showed (Figure 6) changes evoking venous thrombosis of left transverse and sigmoid sinuses, straight sinus, vein of Galen, internal cerebral and basal veins with extensive venous infarcts at the level of bilateral basal ganglia and a left temporo-occipital subcortical area.

She was admitted to the intensive care unit for continuous monitoring and started receiving anticoagulant and supportive treatment represented by continuous intravenous unfractionated heparin with flow adapted to the APTT value, isotonic fluids, vitamin therapy, physical therapy, pneumatic compression, and empiric antiviral therapy until encephalitis was excluded.

On the first day after admission, the patient′s clinical condition deteriorated. She became febrile, presented episodes of drowsiness alternating with spontaneous wakefulness and psychomotor agitation, involuntary hyperextension movements at the thoracic level, bilateral grasping, and plantar clonus bilaterally, and she was only reacting to nociceptive stimuli. The ophthalmological examination ruled out papilledema. A lumbar puncture was performed, and the result was negative for bacteria and viruses. The thrombophilia profile tests were requested and were found to be positive for: homozygous V H129 R mutation, heterozygous PAI1 4G/5G, and homozygous MTHFR A 1289C mutation.

**Figure 7:**
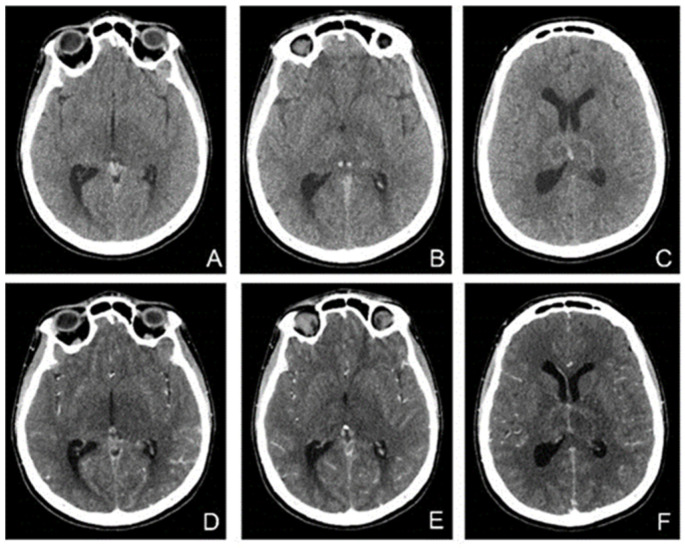
CT without contrast (**A**–**C**) and with (**D**–**F**) contrast enhancement shows hyperdensities in the Galen vein and internal cerebral veins with nonhomogeneous thalamic nuclei. After contrast enhancement, there was a slight enhancement in the veins and hypoperfusion of the thalamic nuclei.

In the following days, the patient′s condition continued to worsen. She did not respond to nociceptive stimuli, spontaneously mobilized her limbs, and had involuntary movements of the jaw. Considering the unfavorable and unpredictable evolution, in agreement with the family, we decided to perform therapeutic abortion. A new brain CT was performed (Figure 7).

Subsequently, with a decreasing Glasgow Coma Scale down to 5 points, the patient needed to be intubated and mechanically ventilated.

After a few days, her condition improved, leading to the patient′s extubation. Then, she was transferred back to the neurology department.

In the meantime, the intravenous heparin therapy was switched to oral anticoagulant therapy—acenocoumarol. Prior to this, brain imaging was repeated and revealed minimal hemorrhagic transformation, but due to the patient’s agitated state during the MRI, we could not achieve enhanced sequences.

Neurological evolution was slowly favorable. Clinical examination before hospital discharge showed a conscious and cooperative patient, without motor and sensory deficits, with memory impairment. 

During hospitalization, the patient could not be cognitively evaluated because she was not always cooperative and had trouble focusing. Later, at the re-evaluation after 2 months, the MMSE and MOCA tests were performed, with a score of 29 and 25 points, respectively, which showed the persistence of a slight cognitive impairment.

Even if the pregnancy was in the first trimester, the association with coagulation disorders led to an increased risk of cerebrovascular phenomena.

## 3. Discussion

There are several risk factors, transient or permanent, that may increase the likelihood of developing cerebral venous thrombosis. Thrombophilia due to antithrombin III or protein C and S deficiency, mutation of factor V Leiden, or hyperhomocysteinemia are permanent risk factors. They are frequently “to blame” in young patients with cerebral venous thrombosis. They can also be incriminated in other situations, such as the ongoing pregnancy or other thrombotic complications such as deep vein thrombosis or pulmonary thromboembolisms. In these cases, and especially if no other associated risk factors are found, it is necessary to perform the thrombophilia genetic profile. It should be noted that mutations in the PAI-1 gene and protein Z are not considered a risk factor for cerebral venous thrombosis [2].

Air pollution is now identified as an independent risk factor for many diseases, including the neurological ones. Air pollution is held responsible for 30% of stroke cases in developing countries [5]. New studies are required to establish the relationship between this new risk factor and CVT.

In our cases, the thrombophilia state was identified through antithrombin III deficiency, respectively, protein C and S deficiency, while two patients had hyperhomocysteinemia. In the case of the pregnant patient, the homozygous mutation V H129 R, the heterozygous PAI-1 4G/5G, and the homozygous mutation MTHFR A 1289C were identified. The latter, being frequently present in the general population, only in association with other prothrombotic factors (especially hyperhomocysteinemia) can be considered of increased risk for CVT. Hyperhomocysteinemia is known to be harmful to endothelial cells and has atherosclerotic and prothrombotic effects. It has been correlated with higher cardiovascular mortality and stroke [6]. 

There are acquired prothrombotic conditions, such as the use of oral contraceptives, smoking, estrogen receptor modulators, pregnancy and puerperium, infections, malignancy, obesity or head trauma [2]. It is also common for the underlying etiology or risk factors not to be identified (37% of older adults), in which case, CVT is considered cryptogenic [2]. In the largest cohort study on CVT (624 patient), the risk factors for an unfavorable outcome were identified and included: male sex, age over 37 years, coma or mental status disorder, intracranial hemorrhage at onset, thrombosis of the deep cerebral venous system, infection of the central nervous system, and malignances [7]. 

It is necessary to state that women during puerperium are more susceptible to hypercoagulability conditions related to this period: pregnancy, caesarean section, massive bleeding, thrombocytosis, fluctuation of intracranial pressure during labor, and pre-eclampsia [8]. During pregnancy, the equilibrium state between the fibrinolytic and hemostatic systems changes in favor of the prothrombotic status to prevent major bleeding during pregnancy and delivery [9]. Thrombocytosis is caused by anemia associated with pregnancy and childbirth, which can cause thrombosis [10], several articles have shown a link between CVT and iron deficiency, with anemia playing a consistent role in the development of CVT [11].

Cesarean delivery increases the risk of venous thromboembolism by three times compared with vaginal delivery [12]. During pregnancy, the women develop resistance to protein C, in addition to losing it through surgery [13]. 

In the first case of the postpartum female, the onset and evolution of CVT were determined by the postpartum prothrombotic condition, aggravated by caesarean section. The complex profile of thrombophilia genetic status, along with the burden of temporary risk factors (anemia, genital infection, obesity, and limitation of mobilization) influenced the response to treatment and the appearance of multiple thrombotic complications.

The infectious state was present in most of the cases exposed, with localized infections being more frequent in our analysis: two cases with an infection in the field of otorhinolaryngology and the postpartum patient having a genital infection. The literature maintains the association with generalized infections as more common in the etiology of CVT. Infectious causes are responsible for only 6–12% of cerebral venous thromboses, with systemic infections being a more frequent cause than local infections (more commonly of the sinuses and mastoid, just as those encountered in our cases) [7].

The onset is frequently associated with signs of cranial hypertension (HIC): variable headaches (intense from the beginning or progressively worse); focal neurological signs or an altered state of consciousness, the latter appearing in up to 61.5% of patients, according to a study from 2007 [14]; and other signs and symptoms such as epileptic seizures (30–40%), papilledema (30–60%), focal motor deficits (30–50%), aphasia (15–20%), mental status disorder (15–25%), coma (5–15%), and movement disorders (rare) [15].

Three patients presented intense and persistent headaches; two of them complained of visual acuity disorder and the patient in the first case had focal onset symptoms similar to a stroke. The initial symptomatology of the pregnant patient (case 4) was with headaches, followed by the alteration of the state of consciousness. This raised the suspicion of cerebral thrombosis, being quickly diagnosed using the angio-MRI. In pregnant patients, the signs and symptoms of thrombosis are generally attributed to their status, as in the case of our patient, and can often cause a delay in the diagnosis and treatment.

MRI with venous TOF sequence is preferred; contrast enhancement is to be avoided in pregnant women with suspected CVT [16]. MRI angiography is superior to CT venography which has low sensitivity for cortical veins. Brain CT is the first intention investigation in which indirect signs such as the chord sign, the dense triangle sign, and the empty delta sign can be observed, and can associate hemorrhagic lesions, focal hypodensities or cerebral edema. In 30% of cases, the CT without enhancement can appear normal [17], as it was in one of the cases presented by us. DSA (digital subtraction angiography) has the highest diagnostic accuracy and is recommended when the basic techniques do not elucidate the diagnosis or when a dural fistula is suspected [3]. The ESO guidelines suggest MRI venography or CT venography to confirm the diagnosis of CVT.

Hemorrhagic transformation can complicate both an arterial stroke and a CVT stroke. In CVT, hemorrhages appeared as a result of increased intracranial pressure due to impaired venous return or the use of heparin used to treat CVT. These hemorrhagic stigmas can also be observed later on the SWI sequence of a brain MRI, with the sequelae of brain parenchyma lesions with hemorrhagic stigma being observed in two of our patients. An imaging feature related to non-hemorrhagic venous infarcts can appear on the follow-up CT, while some of the non-hemorrhagic lesions may disappear, a phenomenon known as “disappearing infarcts” [2].

The presence of the hemorrhagic lesions from the onset, in the first case, delayed the initiation of anticoagulant treatment in the therapeutic dose, putting in balance the risks and benefits, until the diagnosis was established. The association of deep venous thrombosis together with cerebral hemorrhage raised problems regarding the anticoagulant regimen, which could be counterbalanced by the enlarging cerebral hematoma.

Regarding the location of the thrombosis, it is specified to preferentially affect the large sinuses or the confluence of sinuses [7]. The most common locations of CVT occurrence according to the International Study on Cerebral Vein and Dural Sinus Thrombosis (ISCVT) are listed as: transverse sinus (86%), superior sagittal sinus (62%), straight sinus (18%), cortical veins (17%), jugular veins (12%), and vein of Galen and internal brain veins (11%) [14]. In general, there is an association between several sinuses or venous structures, an aspect also observed in our analysis. A study from 2019 mentions the involvement of multiple venous sinuses in 76% of cases, with left heart attacks being twice as frequent as right ones (36% versus 18%) [18]. In the vast majority of people, the cerebral veins on the right side are dominant; hence, an obstruction of the right transverse sinus will have a greater clinical impact [3].

The same reviews mention hemorrhagic transformation in 17.3% of CVT cases and 3.8% in intraparenchymal hemorrhage. Extensive vascular damage as well as the presence of bleeding worsens the prognosis through the severity of intracranial hypertension and the increased risk of complications.

The main objective of the treatment of cerebral venous thrombosis is to stop the progression of the thrombotic phenomenon with the restoration of the flow in the venous system and the prevention of recurrences. For this purpose, the anticoagulant treatment options differ and one can choose between continuous unfractionated heparin (UFH) or subcutaneous low-molecular-weight heparin (Enoxaparin) twice a day [19]. UFH requires dose adjustments based on APTT, this being short-lived, and the effect can be reversed with protamine sulfate. Meanwhile, the effect of Enoxaparin can be reversed only partially, and in a patient with severe renal failure, it is contra-indicated. Thus, the European Stroke Association Guideline recommends the use of enoxaparin instead of UFH, excluding situations in which the patient has renal failure or is likely to require neurosurgical intervention in the near future, or if the patient is pregnant or in the postpartum period [20].

The issue of using new oral anticoagulants for chronic post-CVT anticoagulation is still being studied [20]. The RE-SPECT CVT trial compared the risks and benefits of using warfarin and dabigatran and found both to be safe and without the recurrence of thromboembolic events [21]. A similar result was also obtained in a comparative study of rivaroxaban versus warfarin published in 2020 [22] after which there is no different significant efficiency and safety. A 2022 meta-analysis mentions that the use of direct-acting oral anticoagulants (DOAC) in CVT is as effective and safe as using vitamin K antagonists, with a better recanalization rate in favor of DOAC, but requires prospective randomized studies for confirmation [23]. In addition, due to their targeted effect in the coagulation cascade, compared to Warfarin, they also have the advantage of a much lower risk of hemorrhagic complications. These observations were mentioned in comparative studies performed on patients with symptomatic deep vein thrombosis [24]. Without imposing dietary restrictions, with a reduced number of drug interactions, a constant therapeutic concentration, and no need for periodic monitoring as required by vitamin K antagonists, DOAC gives them multiple advantages and, through that, increases the compliance as well. In addition, the existence of a specific medication to reverse the effect of DOACs increases their safety even in the event of acute complications such as hemorrhage, emergency surgeries, or ischemic stroke in a therapeutic window [25]. New clinical trials are needed to analyze their benefits and risks in cerebral venous thrombosis, thus contributing to the development of the next guidelines.

However, the European Stroke Association Guideline, published before the results of these studies, does not recommend the use of oral anticoagulants (thrombin or factor Xa inhibitors) for the prevention or treatment of the acute phase of CVT (ESO 2017). The use of a vitamin K antagonist is recommended for 3 to 12 months after acute CVT [19].

Regarding hemorrhagic complications of CVT, nowadays, the same guideline recommends the initiation of anticoagulant treatment in the therapeutic dose, regardless of the presence of bleeding, with close clinical and paraclinical monitoring [20]. 

We noticed that although the patients were under effective anticoagulant treatment, three of them presented clinical fluctuations, different thrombosis ages, and extracerebral thrombotic events. A possible explanation is the summation of thrombophilia risk factors (possibly in association with homocysteine increase) in addition to an inflammatory response to an infectious background that could lead to neurologic fluctuations.

The first episode of CVT with transient risk factors requires ACO treatment for three to six months (six to twelve months if cryptogenic). The presence of properly diagnosed thrombophilia (at a distance from temporary conditions that can change the real values) requires permanent anticoagulant treatment.

In the case of a patient with permanent risk factors (genetic thrombophilia), the oral anticoagulation treatment is administered continuously. Case number four is on a two-month course of anticoagulant treatment and was to return in three months for a new clinical imaging and thrombophilia reassessment to decide the indication of anticoagulants.

The vast majority of patients require anticoagulant treatment for a minimum of 3 to a maximum of 12 months, but in the four cases presented, three of them required chronic anticoagulant treatment due to the type of thrombophilia and the risk associated with it [15]. 

The following studies, as well as the following guide regarding CVT, should offer recommendations regarding the possibility of using the new oral anticoagulants, taking into account the aspect identified in the three cases (the need for permanent anticoagulation).

Additionally, future studies/guides should take into account the conclusions of the study led by Mrs. Aguiar de Sousa et al. (2020), namely the fact that venous recanalization begins in the first 8 days and that the age of the patient must also be taken into account, so as to reduce the hemorrhagic risks associated with anticoagulant therapy [26]. Pathogenic therapy is combined with symptomatic and risk factor therapy, which imposes the long-term therapy of CVT. It should be noted that anticonvulsant therapy should not be recommended as a preventive measure and if it is necessary, it is recommended to choose a medication that does not interfere with the anticoagulant medication. These patients will be clinically and paraclinical monitored under anticonvulsant treatment for a minimum of one year [27].

Complications are relatively rare and can be grouped into acute or chronic. Acute complications are venous infarction and/or hemorrhage, subarachnoid hemorrhage, pulmonary thromboembolism, motor or language deficits, rapid progressive deterioration of mental status or coma, or in cavernous sinus involvement, Korsakoff-like amnestic syndrome with confabulation, bilateral temporal lobe infarction, inadequate antidiuretic hormone secretion, and blindness. Hemorrhagic complications were present in two of the patients, alongside the rapid alteration of the state of consciousness in case 4: that of the pregnant woman who required the rapid intervention of intensive care.

The most common chronic complications are arterio-venous fistulas, epilepsy, and psychiatric complications (cognitive impairments, depression, and anxiety) [4,28].

Neurological deficits, such as paresis or slurred speech, can also persist if the venous damage has also caused damage to the brain parenchyma, which deprives these functions.

Regarding the long-term complications of cerebral venous thrombosis, we also draw attention to the fact that depression can be a chronic complication of CVT [4] and various other behavioral disorders can occur, depending on the site, resulting in a significant burden for the patient and their family if not treated and closely monitored. Although in most cases neurological recovery after cerebral thrombosis is very good, depression and anxiety occur frequently between 1/3 and 2/3 of the cases [29]. 

Post-stroke depression, an emotional incontinence, is strongly influenced by lesion location, probably associated with the chemical neuroanatomy related to the frontal/temporal lobe–basal ganglia–ventral brainstem circuitry. Michaela C et al. reported that hyperhomocysteinemia is correlated with an increased risk of clinical depression. Depression is the most prevalent psychiatric complication among stroke survivors. Post-stroke depression is correlated with reduced motor function and poorer outcome [30].

Abulia, executive deficits, and amnesia may result from thrombosis of the deep venous system, causing bilateral thalamic infarcts. Recovery is variable, but memory deficits, behavioral problems, or executive deficits may persist [31]. Regarding case four, we noticed behavioral disorders such as quickly abandoning a just-started activity and lack of patience. Patients should be encouraged to return to previous occupations and hobbies.

In case one, the patient with the hemorrhagic left parietal lesion, the depression after CVT was severe without finding a correlation between the affected brain aria and its specific syndrome. We mention that the patient had no previous depression. A study on depression and its relation to lesion location after stroke reported an association between lesions on the right hemisphere, particularly the anterior region and depression [31]. Another 2010 study of six patients with depression and suicide attempts after stroke found that five of them had moderate neurological deficits; moreover, in five cases, lesions were identified at the temporo-parietal cortex level, with dominance slightly on the left side (three versus two), but without statistical significance. There was no evidence of hemorrhagic transformation in any of the cases [32].

The patient with a longer evolution of CVT at the level of the cavernous sinuses developed depression and later focal convulsions. 

One in ten patients may have late seizures after a CVT [33]. According to the International Epilepsy League, we can diagnose epilepsy after one unprovoked seizure if there are specific conditions that imply a 60% or higher risk of developing subsequent epilepsy, which was the case in our third patient, who presented not only interictal discharge on their EEG, but also a favorable factor—CVT [34].

In the last case reported with the dominant acute symptomatology of the altered state of consciousness and memory disorders, we observed an improvement in cognitive deficits at the evaluation after 2 months. A psychological assessment of each type of cognitive function will bring us additional data in the future. The long-term follow-up of this patient should particularly take notice of focusing difficulties and behavioral impairment.

We thought it important to draw attention to depression as a potentially life-threatening complication of CVT. The identification of additional risk factors and the rigorous neuropsychiatric monitoring of these patients are essential in the ad vitam prognosis.

Further investigations studying the pathophysiological correlations between cerebral venous thrombosis lesions and psychiatric complications could lead to their prevention and appropriate management.

The prognosis of patients with CVT is generally favorable and depends on the speed of diagnosis and treatment. Complete clinical recovery is achieved in approximately 75% of cases, but there is also the risk of residual neurological deficit or death (15%) [7].

The CVT-GS grading scale can be used to calculate outcome prediction after a CVT (Table 1). In our cases, the score was as follows: case 1–5 points, moderate CVT with a 30-day mortality rate of 9.9%; case 2–2 points, mild CV with a 30-day mortality rate of 0.4%; case 3–0 points, mild CVT with a 30-day mortality rate of 0.4%; and case 4–11 points, severe CVT with 30-day mortality of 61.4% [35].

Negative prognostic factors are represented by intracranial hemorrhage, male sex, CVT outside of pregnancy, puerperium, or the use of oral contraceptives, and were present in three of our patients. In the literature, it is mentioned that in 85% of cases, they have at least one risk factor present [36,37]. 

The risk of recurrence of any other thrombotic event after a CVT is approximately 6.5%, and most recurrences appear in the first year [38] after the thrombotic event. Men tend to have a worse recovery compared to women: 81% of women will have a full recovery, while men only 71% of them. Severe and mild thrombophilia are the most important relapse risk factors (four times higher). Mild thrombophilia is considered to be one of the following: heterozygous factor V Leiden and prothrombin G20210A mutation, while protein C, S and antithrombin III deficiencies, antiphospholipid antibodies, and homozygous factor V Leiden are considered to be a severe form of thrombophilia [29]. The risk of recurrence was also increased in patients who stopped anticoagulant treatment early [39]. 

The 2017 ESO guideline does not recommend screening for thrombophilia in order to prevent recurrences or improve the prognosis; yet, in patients with a high probability of having thrombophilia, it can be considered. This category includes a patient’s high pre-test probability to suffer from a severe form of it, young age at the onset of CVT, no other risk factors, and personal or family history of a form of venous thrombosis. 

Based on the available evidence, CVT is not a contraindication for future pregnancies, but if a prothrombotic condition or a previous thrombotic event exists, antithrombotic prophylaxis is necessary during pregnancy and in the puerperium period.

## 4. Conclusions

The association of multiple risk factors in the case of cerebral venous thrombosis determines a negative prognosis and unexpected late complications. The presence of bleeding at the onset makes it difficult to establish a quick diagnosis and the therapeutic decision balances risk–benefit, requiring close monitoring. An anticoagulant treatment, even in the therapeutic dose, may not offer safe protection for the development or occurrence of other thrombotic complications. The recurrences and severity of CVT encountered in real life can determine new directions of study regarding the identification of additional risk factors involved as well as conditions that determine the choice of a certain type of anticoagulant. The successful management of CVT depends on the rapidity of the etiological diagnosis and the promptness of the therapy in the acute phase, coupled with the careful monitoring of the patient to prevent or intervene immediately in case of acute or chronic complications. Epilepsy, depression, or cognitive impairment are complications in the evolution of patients requiring close monitoring to prevent unpredictable behavior.

## Figures and Tables

**Table 1:** CVT-GCS Scale—Risk of mortality at 30 days calculated for each case presented.

CVT-GS Scale	Case 1	Case 2	Case 3	Case 4
Parenchymal lesion > 6cm—3pt	3	0	0	3
Bilateral Babinski sign—3pt	0	0	0	3
Male sex—2pt	0	2	0	0
Parenchymal hemorrhage—2pt	2	0	0	2
Level of consciousness - Coma 3pt - Stupor 2pt - Alert 0pt	0	0	0	3
**TOTAL**	**5**	**2**	**0**	**11**
**30-day case fatality**	**9.9%**	**0.4%**	**0.4%**	**61.4%**

## Data Availability

Third-party data restrictions apply to the availability of these data. The data were obtained from “Dr. Carol Davila” Central Military Emergency University Hospital Bucharest and are available from the authors with the permission of the Institutional Ethics Committee of Clinical Studies of the “Dr. Carol Davila” Central Military Emergency University Hospital Bucharest.
